# Codon Optimisation Is Key for Pernisine Expression in *Escherichia coli*

**DOI:** 10.1371/journal.pone.0123288

**Published:** 2015-04-09

**Authors:** Marko Šnajder, Marko Mihelič, Dušan Turk, Nataša Poklar Ulrih

**Affiliations:** 1 Biotechnical Faculty, University of Ljubljana, Ljubljana, Slovenia; 2 Centre of Excellence for Integrated Approaches in Chemistry and Biology (CipKeBiP), Ljubljana, Slovenia; 3 Institute Jozef Stefan, Ljubljana, Slovenia; Aligarh Muslim University, INDIA

## Abstract

**Background:**

Pernisine is an extracellular serine protease from the hyperthermophilic *Archaeon Aeropyrum pernix* K1. Low yields from the natural host and expression problems in heterologous hosts have limited the potential applications of pernisine in industry.

**Methodology/ Principal Findings:**

The challenges of pernisine overexpression in *Escherichia coli* were overcome by codon preference optimisation and *de-novo* DNA synthesis. The following forms of the *pernisine* gene were cloned into the pMCSGx series of vectors and expressed in *E*. *coli* cells: wild-type (*pernisine^wt^*), codon-optimised (*pernisine^co^*), and codon-optimised with a S355A mutation of a predicted active site (*pernisine^S355Aco^*). The fusion-tagged pernisines were purified using fast protein liquid chromatography equipped with Ni^2+^ chelate and gel filtration chromatography columns. The identities of the resultant proteins were confirmed with N-terminal sequencing, tandem mass spectrometry analysis, and immunodetection. Pernisine^wt^ was not expressed in *E*. *coli* at detectable levels, while pernisine^co^ and pernisine^S355Aco^ were expressed and purified as 55-kDa proforms with yields of around 10 mg per litre *E*. *coli* culture. After heat activation of purified pernisine, the proteolytic activity of the mature pernisine^co^ was confirmed using zymography, at a molecular weight of 36 kDa, while the mutant pernisine^S355Aco^ remained inactive. Enzymatic performances of pernisine evaluated under different temperatures and pHs demonstrate that the optimal enzymatic activity of the recombinant pernisine is *ca*. 100°C and pH 7.0, respectively.

**Conclusions/ Significance:**

These data demonstrate that codon optimisation is crucial for pernisine overexpression in *E*. *coli*, and that the proposed catalytic Ser355 has an important role in pernisine activity, but not in its activation process. Pernisine is activated by autoproteolytical cleavage of its N-terminal proregion. We have also confirmed that the recombinant pernisine retains the characteristics of native pernisine, as a calcium modulated thermostable serine protease.

## Introduction

The thermostable serine protease pernisine is potentially useful to various industries, from the cleaning industry to medical fields, where high temperatures or harsh conditions are encountered (e.g., with denaturants, reductants or detergents) [1,2]. In 2012, we demonstrated enzymatic degradation of protein aggregates by pernisine, such as for infective prions (PrP^Sc^) from different origins (i.e., mouse, bovine, deer, human). More recently, the Archaeon *Thermococcous kodakaraensis*, which is closely related to *Aeropyrum pernix*, was shown to degrade PrP^Sc^ through its subtilisin (Tk-subtilisin) activity and through its *subtilisin*-like serine protease [3,4]. However, PrP^Sc^ is not completely degraded by mesophile proteases, and thus at high temperatures, thermostable proteases like pernisine can be exploited [2].

Pernisine is a gene product of *A*. *pernix* K1, which lives in hot solphataric vents at temperatures of around 100°C [5]. Based on gene sequence alignment, pernisine is a subtilisin-like serine protease (i.e., a subtilase) with the catalytic triad of Asp149, His184 and Ser355 [6]. The subtilases are generally synthesised as inactive precursors that contain the signal sequence followed by a proregion at the N-terminus [6]. For native pernisine, the signal sequence and proregion were predicted for the first 24 amino acids (aa) and 92 aa, respectively [2]. Problems of obtaining high amounts of native or recombinant pernisine have hampered its biochemical characterisation and its potential use in industry. The final yield of purified pernisine has been reported as ca. 0.5 mg per litre of *A*. *pernix* culture broth, which is below that acceptable for its industrial production [2]. Additionally, the natural host *A*. *pernix* produces other extracellular proteases, such as protease I [7]. Indeed, the overexpression of functional, thermostable enzymes in mesophilic hosts like *E*. *coli* can be challenging [8].

Heterologous expression systems are often used to produce higher yields of proteins compared to the natural host. Although there are a variety of bacteria, Archaea and eukaryote expression systems, the most common and preferred expression system host remains *E*. *coli* [9,10]. The advantages of *E*. *coli* are its fast growth, relatively high protein yields, low cost, high diversity of cloning vectors, easy handling, and versatile strains for the production of demanding target proteins. However, like other expression systems, *E*. *coli* has its drawbacks, especially for the production of target proteins of distant origin that might include posttranslation modifications, toxic influences, or rare codons for the host [9,11]. These challenges can be overcome by glycosylation system transfer [12], tighter control of the expression system or different promoters [9], and DNA sequence manipulation with codon-optimisation strategies [8,13,14,15]. Indeed, over the last decade, the use of codon-optimised genes in industrial biotechnology has reduced the cost of protein production, through improved protein expression [11].

The aim of the present study was to define an efficient *E*. *coli* expression system for the production of functional pernisine, to evaluate the effects of mutation of the proposed pernisine catalytically active Ser355, and to define the pernisine activation process.

## Materials and Methods

### Codon optimisation (pernisine^co^, pernisine^S355Aco^)

The *pernisine* gene (1293 bp) that was inferred from homology studies was codon optimised (*pernisine* ^*co*^) and synthesised for an *E*. *coli* expression system (Genscript). In all, 327 of 1293 nucleotides were changed, without changing the translated aa sequence, except for the introduction of the mutation of S355A, to give *pernisine* ^*S355Aco*^.

### Assembly of pMCSGx expression vectors (x = 7, 9, 10)

*Aeropyrum pernix* was cultivated as previously described [16], and its genomic DNA (gDNA) was isolated using gDNA isolation kits (Sigma). This *A*. *pernix* gDNA was used as the template for the wild-type pernisine (*pernisine* ^*wt*^). The *pernisine*, *pernisine* ^*co*^ and *pernisine* ^*S355Aco*^ genes were obtained using polymerase chain reaction (PCR), and cloned according to the relevant instruction manuals [17]. Briefly, the PCR products of these *pernisine* genes were amplified using sense and antisense primers: wild-type (5`-TACTTCCAATCCAATGCCGCAGCAGGATCGGCGGCTGGGGCTAG-3`, 5`-TTATCCACTTCCAATGTTAGCTTGAGACGGCAGTCTGCAC-3`) and codon-optimised (5`-TACTTCCAATCCAATGCCGCAGCAGGTACGAAAATCGCCGCTATCGC-3`, 5`-TTATCCACTTCCAATGTTAACTGGAGACAGCCGTTTGGACAG-3`). The treatment of the PCR products with T4 DNA polymerase in the presence of dCTP generated 15 nucleotides with long single-strand overhangs. Conversely, the treatment of the previously linearised pMCSGx vectors with the restriction enzyme *Ssp*I followed by T4 DNA polymerase in the presence of dGTP created the complementary overhangs. In the next step, the plasmid and the PCR products were linked in the annealing process. The ligation-independent cloned constructs were transformed into competent DH5α cells, which were grown in Luria-Bertani (LB) medium to produce larger quantities of the vectors. Three different constructs with different tags for each gene were constructed.

### Strains, expression of pernisine^wt^, pernisine^co^, pernisine^S355Aco^, and purification

The purified expression vectors were transformed into the competent BL21(DE3) *E*. *coli* strain. In addition, the *pernisine* ^*wt*^-containing vectors were transformed into the BL21(DE3)pLysE, BL21(DE3)pMAGIC [F^–^ompT hsdS(rB^–^mB^–^) dcm^+^Tet^r^gal λ(DE3) endA Hte] and BL21-CodonPlus(DE3)RIL [F^–^ompT hsdS(rB^–^mB^–^) dcm^+^Tet^r^gal λ(DE3) endA Hte [argU ileY leuW Cam^r^]

*E*. *coli* strains and plated in the appropriate selection medium. The selected transformants were grown as a mini-scale batch (10 ml LB medium) and the plasmids were purified using GenElute plasmid miniprep kits (Sigma). The DNA was sequenced (Macrogene), and the transformants with confirmed pernisine DNA were used for large-scale expression (4.0 L LB medium).

A single colony was cultivated overnight at 37°C in 25 ml LB medium supplemented with the appropriate antibiotic, under constant agitation at 240 rpm. The next day, 475 ml fresh LB medium containing the appropriate antibiotic was added to 25 ml of the overnight culture. When the cells reached an optical density at 600 nm (OD_600_) of 0.6 to 0.8, expression was induced with 1 mM isopropyl β-D-1-thiogalactopyranoside. The culture growth times after this induction ranged from 1 h to 4 h, as optimised initially by the detection of pernisine on dot blots. The cells were centrifuged (6,000x *g*, 20 min, 4°C) and resuspended in 25 ml lysis buffer (30 mM Tris-HCl, 0.3 M NaCl, 1 mg ml^-1^ lysozyme, pH 7.5). The cells were then lysed by sonication (amplitude 40%; 10 s on, 10 s off; 120 s; VCX 750 by Sonics), and centrifuged (19,000x *g*, 20 min, 4°C). The supernatants were used for analysis and purification of pernisine. The pellets were resuspended in 4 M urea and subjected to SDS-PAGE, for determination of the insoluble pernisine fraction.

N-terminal His_6_-tagged pernisine was purified using Ni^2+^-Sepharose 6 FF columns (GE Healthcare), followed by size exclusion chromatography using a HiLoad 16/60 Superdex 200 preparative grade column (GE Healthcare). The unbound samples were washed out with 20 column volumes of binding buffer (20 mM Na_2_HPO_4_, 0.5 M NaCl, 20 mM imidazole, pH 7.4), and the bound samples were eluted with the same buffer containing 500 mM imidazole. The eluted proteins were applied directly onto the size exclusion column, which was equilibrated with 30 mM Tris HCl, 0.3 M NaCl, pH 7.4. The pernisine fractions were collected, dialysed (SPECTRA/POR, MWCO 8–10 kDa) in 10 mM HEPES, pH 8.0, for 4 h, and lyophilised (Christ alpha 1-2LD Plus, Germany). All of the purification procedures were performed at 4°C.

### Activation of recombinant pernisine

Recombinant pernisine (1 mg ml^-1^) was dissolved in activation buffer (10 mM HEPES, 1 mM CaCl_2,_ pH 8.0) and heat activated in 100 μl aliquots in PCR tubes, at 90°C for 1 h; this activated recombinant pernisine was then used for proteolytic assays, unless otherwise specified. Preliminary tests for the activation were performed, with the recombinant pernisine (1 mg ml^-1^) incubated in activation buffer at different temperatures (60, 80, 90°C) for specific times (0, 15, 30, 60, 120 min). Immediately after these incubations, azocasein assays were carried out to determine the proteolytic activities of the samples (not shown). The specified time when the proteolytic activity at each temperature reached maximum was considered as full conversion of the proform of recombinant pernisine to the mature form of pernisine. The optimal activation conditions chosen were 1 h at 90°C.

### SDS-PAGE, Western blotting and dot blots

Protein samples (10 μg) were analysed by SDS-PAGE using 12% polyacrylamide gels, and visualised using Coommassie brilliant blue staining. Cell lysates were normalised to cell density. For the Western blotting, the proteins were electrotransferred to nitrocellulose membranes. The membranes were blocked with 5% (w/v) non-fat dry milk in Tris-buffered saline with 0.05% (v/v) Tween 20 (TBST) at room temperature for 1 h. The His_6_-tagged pernisine was detected using rabbit polyclonal anti-histidine antibodies diluted 1:1,000, with incubation at room temperature for 1 h. The bound antibodies were detected with horseradish-peroxidase-conjugated goat anti-rabbit IgG antibodies (dilution, 1:2,000). Visualisation was performed using the ELC detection reagent, according to the manufacturer instructions (GE Healthcare). The dot blot analysis was carried out using anti-His_5_ antibodies (Qiagen) diluted 1:1,000, according to manufacturer instructions. These dot blots for the different expression times for pernisine after the induction were quantified using the ImageJ software.

### N-terminal sequencing

After separation by SDS-PAGE, the proteins were transferred onto PVDF membranes (Bio-Rad). These membranes were rinsed with Milli-Q water, stained with Ponceau S (Sigma) for 2–3 min, and destained with several changes of Milli-Q water. Purified His_6_-tag pernisine was cleaved with tobacco etch virus (TEV) protease, and subjected to SDS-PAGE. The excised tagless pernisine band was subjected to N-terminal sequencing, by automatic degradation in a Procise 492A protein sequencer (PE Applied Biosystems) at the Jozef Stefan Institute, Slovenia.

### Tandem mass spectrometry

**Fig 1:**
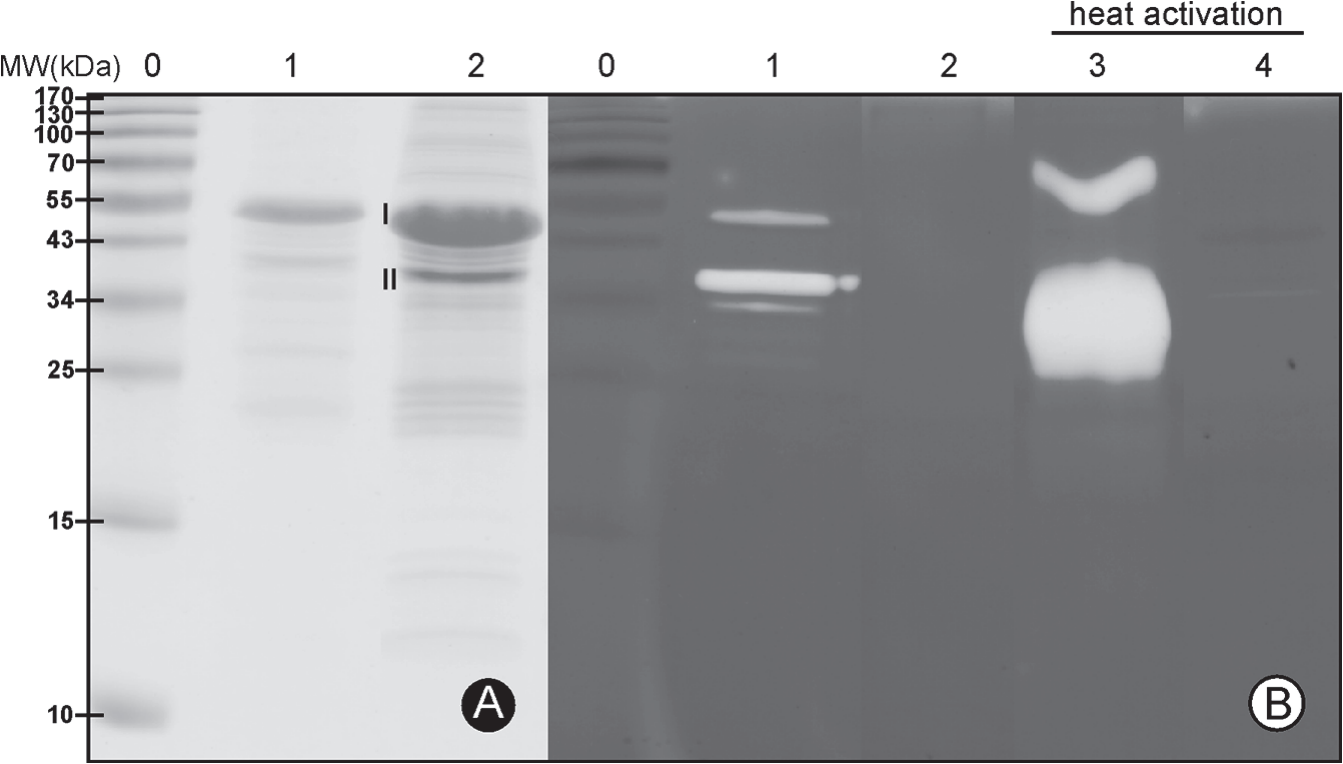
SDS-PAGE analysis and zymography of purified pernisine^co^ and pernisine^S355Aco^. Representative gels of the purified pernisines, following electrophoresis on standard 12% SDS-PAGE (A) and on 12% SDS-PAGE with casein as substrate (B) for the zymography activity (4 h at 80°C). Staining was with Coomassie blue dye. Lanes 0, protein MW markers (indicated left); lanes 1, recombinant pernisine^co^; lanes 2, recombinant pernisine^S355Aco^; lanes 3 and 4, heat-activated pernisine^co^ and pernisine^S355Aco^. Selected protein bands of pernisine^co^ that were analysed by MS/MS are marked as I and II, (see also S3 and S4 Figs). *protein load of pernisine^**S355Aco**^ is three times higher than pernisine^co^.

Marked bands I and II shown in Fig 1A were cut out of the SDS-PAGE. The reagents were prepared in 100 mM ammonium bicarbonate buffer. The protein samples were reduced using 10 mM dithiothreitol at 56°C for 30 min, and alkylated with 55 mM iodacetamide at room temperature for 20 min. The gel pieces were transferred to glass tubes (300 μL) and 20–30 μL 3 M HCl was added. These tubes were placed inside tubes containing 700 μL water and microwaved for 10 min. Afterwards, the supernatant was removed from the glass tubes and desalted directly on Oasis HLB Elution Plate (Waters), according to the manufacturer instructions. The samples were eluted (50 μL) and dried in a SpeedVac centrifuge (Eppendorf). The dried samples were dissolved in 10 μL reconstitution buffer (water: acetonitrile [96:4, v/v] in 0.1% formic acid), and analysed at the European Molecular Biology Laboratory (EMBL), Germany.

The peptides were separated using a nanoAcquity ultra-performance liquid chromatography system (Waters) fitted with a trapping column (nanoAcquity Symmetry C_18_) and an analytical column (nanoAcquity BEH C_18_). Solvent A was 0.1% formic acid in water, and solvent B was 0.1% formic acid in acetonitrile. The samples (8 μL) were loaded onto the trapping column with 5 μL min^-1^ solvent A. The peptides were eluted via the analytical column with a flow rate of 0.3 μL min^-1^. During the elution step, solvent B increased as a linear gradient from 3% to 10% over the first 5 min, and then to 40% over the next 10 min. The peptides were introduced into the mass spectrometer (Orbitrap Velos Pro; Thermo Scientific) using a Pico-Tip Emitter tip (New Objective), with a spray voltage of 2.2 kV applied. The capillary temperature was set to 300°C. Full scan mass spectra with a mass range of 300–1700 *m/z* were acquired in profile mode in the Fourier transform with a resolution of 30,000. The most intense ions (up to 15) from the full scan mass spectra were selected for sequencing in the linear trap quadropole mass spectrometer. A normalised collision energy of 40% was used, and fragmentation was performed after accumulation of 3 ×10^4^ ions, or after a filling time of 100 ms for each precursor ion (as whichever occurred first). The tandem mass spectrometry (MS/MS) data were acquired in centroid mode. Only multiply charged precursor ions (2+, 3+, 4+) were selected for the MS/MS.

The MaxQuant software was used to filter the data and to create the. mgf files that were needed for searching in MASCOT (Matrix Science). The data were searched against a species-specific (*Aeropyrum pernix* K1) Uniprot database. The data were searched with the following modifications: Carbamidomethyl (C) (Fixed) and Oxidation (M) (Variable). The mass error tolerance for the full scan mass spectra was set at 20 ppm, and for the MS/MS spectra, at 0.5 Da. A search with no enzyme was used. The termini were postulated based on peptide ladders of increasing aa length, with all either starting or ending at the same residue (for the N- and C-termini, respectively).

### Protease activity

The protein concentrations were determined spectrophotometrically using the extinction coefficient of $\varepsilon_{280nm}^{1\%}$ = 60,850 M^-1^ cm^-1^ for pernisine with its proregion, and $\varepsilon_{280nm}^{1\%}$ = 57,870 M^-1^ cm^-1^ for the mature pernisine. Alternatively, they were determined by the Bradford method [18], using BioRad Protein Assay (BioRad) with bovine serum albumin as standard.

To determine the qualitative proteolytic activity of the recombinant pernisine, zymography procedures with standard SDS-PAGE were used, as described previously [2]. Briefly, the samples were applied in duplicates onto 12% SDS-PAGE gels without and with 0.1% (w/v) casein (Sigma Aldrich) and electrophoresed (125 V, 70 min). The gels with added casein were transferred into 2.5% Triton X-100 for 1 h, washed twice with buffer (50 mM Tris-HCl, 1 mM CaCl_2_, pH 8.0), and incubated in the same buffer at 80°C for 4 h. The proteolytic activity was visualised as clear bands on the gels, against a blue background, using Coomassie brilliant blue staining. The SDS-PAGE gels without casein were stained immediately after electrophoresis.

To characterise the recombinant pernisine, azocasein assays were used, as described previously [2], with addition of the pernisine activation step. The samples were assayed as triplicates and the standard errors calculated. Initially, the optimum proteolytic activities of the recombinant pernisine in the presence of different CaCl_2_ concentrations (0–32 mM) were examined. Then, the effects of ionic strength on the pernisine activity were investigated, as different NaCl concentrations (0–500 mM). To define the optimum pernisine activity, standard azocasein assays were conducted at different temperatures from 40°C to 120°C, and at different pHs from pH 2 to pH 12. The buffers used were: pH 2 to pH 4, 50 mM glycine-HCl; pH 6 to pH 8, 50 mM HEPES; and pH 9 to pH 13, 50 mM glycine-NaOH. The pH at each incubated temperature was calculated according to the dpH/dT correction coefficient [19]. Then, a three-dimensional graph of the temperature and pH dependence against the pernisine relative activity was plotted using the OriginPro 8 programme. In the same way, the thermostability of the pernisine was evaluated using standard azocasein assays at different temperatures (40, 80, 110, 120°C) and incubation times (0.1, 1, 2, 4 h) in 50 mM Tris-HCl, pH 8.0 with 1 mM CaCl_2_.

To evaluate the effects of inhibitors, reductants, denaturants and detergent on pernisine proteolytic activity, the samples in the reaction mixtures were incubated at room temperature for 10 min prior to the azocasein assays. The inhibitors studied were ethylenediaminetetraacetic acid (EDTA; 1, 5 mM), ethylene glycol-bis(b-aminoethyl ether)-*N*,*N*,*N*,*N*-tetraacetic acid (EGTA; 1, 5 mM), phenylmethylsulphonyl fluoride (PMSF; 1, 10 mM) and iodoacetamide (IAA; 1, 10 mM). The reductants were dithiothreitol (DTT; 1, 5 mM) and 2-mercaptoethanol (1%, 5%), the denaturants were guanidinium hydrochloride (GdnHCl; 1, 4 M) and urea (1, 4 M), and the detergent was SDS (0.1%, 3.0%).

## Results and Discussion

### Design and cloning of the wild-type and synthetic pernisine genes

The efficiency of pernisine overexpression in the BL21(DE3) *E*. *coli* cells was compared between the wild-type and codon-optimised pernisine sequences. The synthetic *pernisine* gene was designed using the GeneOptimiser algorithm (Genscript) and synthesised by Genscript. Moreover, the predicted catalytic serine at site 355 was mutated into alanine (S355A) to analyse the enzymatic activity that then remained. The *pernisine* gene consists of 1293 bp (European Molecular Biology Laboratory: BAA79718.2), and it was amplified using specified primers and the gDNA of *A*. *pernix* K1 (i.e., *pernisine* ^*wt*^) or the synthetic codon-optimised genes (i.e., *pernisine* ^*co*^, *pernisine* ^*S355Aco*^) as templates. This codon optimisation replaced the rare codons in the *pernisine* ^*wt*^ gene with more frequent codons, as given in the S1 Table, while the aa sequence remained unchanged. The GC nucleotide content remained the same, at 57%. Altogether, 25.3% of the nucleotides in the DNA sequence were changed (S1 Fig). With the *de-novo* synthesis, the mRNA stability was improved, and the unfavourable mRNA structures and rare codons were reduced. Heterologous expression of rare codon-containing genes is likely to exhaust the endogenous pools of the analogous tRNAs and lead to growth inhibition, premature termination of transcription and/or translation, decreased mRNA stability, and increased frameshifts, deletions and misincorporations. Similar techniques of improved overexpression have been shown for some other proteins [8,10,20,21].

High-throughput cloning of the *pernisine* ^*wt*^ and *pernisine* ^*co*^ sequences in the pMCSGx series of vectors incorporates the N-terminal tags, followed by a TEV cleavage site (His_6_-TEV, His_6_-maltose binding protein [MBP]-TEV, His_6_-glutatione S-transferase [GST]-TEV). Agarose electrophoresis revealed that the lengths of the amplified *pernisine* ^*wt*^ and *pernisine* ^*co*^ corresponded to the expected *ca*. 1,300 bp. Linearised pMCSGx (x = 7, 9, 10) vectors were seen at the expected lengths of *ca*. 5300, *ca*. 6000 and *ca*. 6300 bp (data not shown).

Specific removal of the tags is an option when there is a TEV cleavage site between the tags and the pernisine [17]. The His_6_ tag was used for simplified purification and detection of pernisine and MBP, with the GST tag to improve solubility [22].

### Overexpression and purification of recombinant pernisine

**Fig 2:**
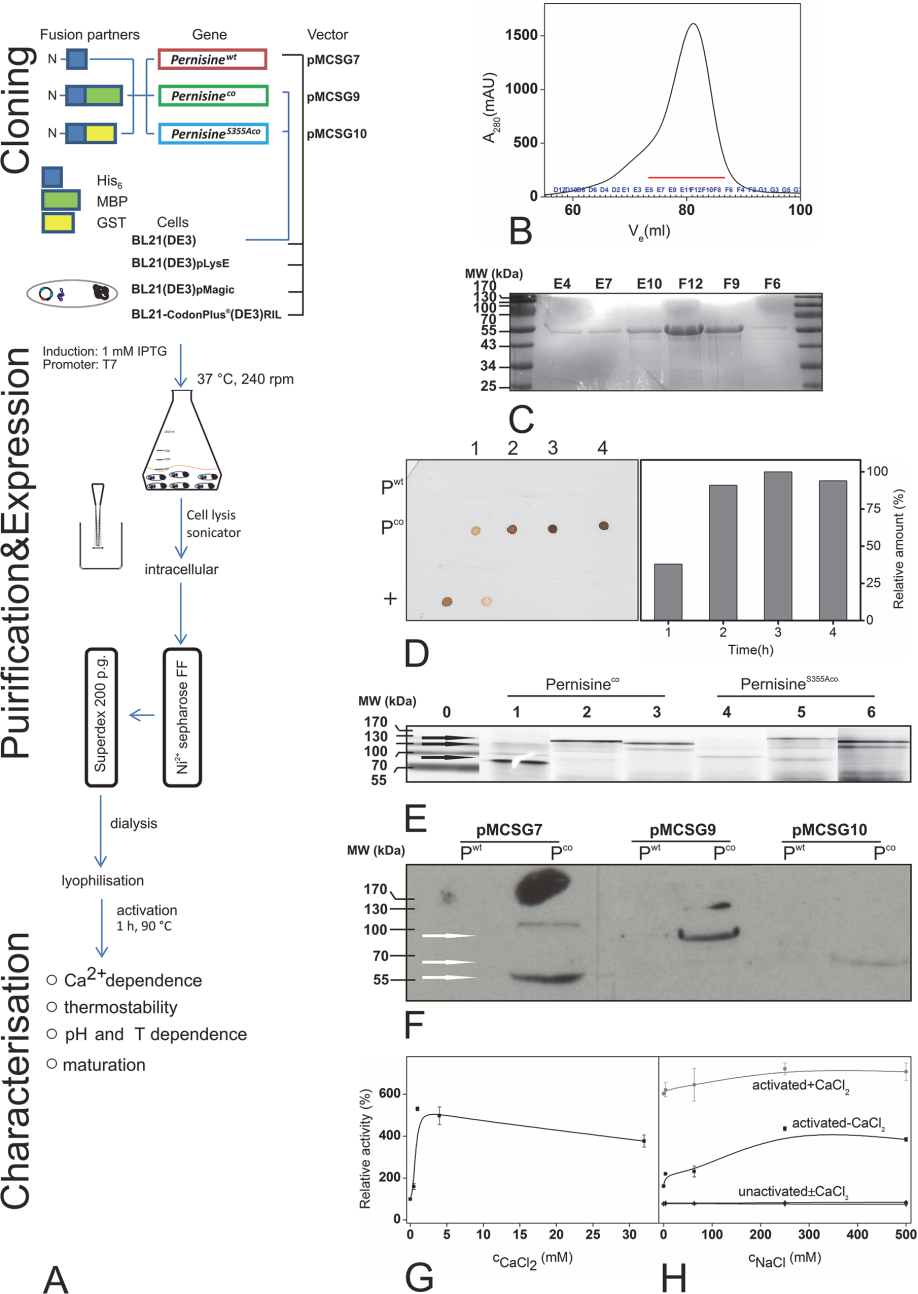
Experimental flowchart and pernisine expression and analysis. (A) Flowchart of the experimental procedures. (B, C) Gel-exchange chromatography of pernisine^co^ in pMCSG7 using BL21(DE3) *E*. *coli* cells (B), and the corresponding SDS-PAGE analysis (C). The red line represents the selected fractions. (D) Time expression analysis after induction (1, 2, 3, 4 h) of *pernisine* ^*wt*^ and *pernisine* ^*co*^ for total cell lysates. Proteins were transferred (dot blot) onto nitrocellulose membranes and His_6_-tagged pernisine was detected with anti-His_5_-tag antibodies. Quantification was done using the ImageJ software (right panel). (E) SDS-PAGE analysis of pernisine^co^ and pernisine^S355Aco^ for total cell lysates of BL21(DE3) *E*. *coli* containing the pMCSGx series of vectors. 1, 4, Pernisine^co/wt^-pMCSG7; 2, 5, Pernisine^co/wt^-pMCSG9; 3, 6, Pernisine^co/wt^-pMCSG10. (F) Immunodetection of pernisine^wt^ and pernisine^co^ for cell lysates containing the pMCSGx series of vectors. Proteins were transferred onto nitrocellulose membranes and His_6_-tagged pernisine was detected using anti-His_6_-tag antibodies. (G, H) Azocasein assays of the purified pernisine^co^, showing effects of CaCl_2_ (G) and NaCl (H). Relative proteolytic activities of activated pernisine^co^ are shown according to the CaCl_2_ concentrations, and to the NaCl concentrations in the presence (grey, dot-dash line) and absence (black line) of 1 mM CaCl_2_. Non-activated pernisine^co^ in the absence of 1 mM CaCl_2_ is also shown (black, dot line). Abbreviations: co-codon-optimised, wt-wild-type.

Various expression strains of *E*. *coli* transformed with the pMCSGx constructs containing the wt or codon-optimised pernisine sequences were tested. The recombinant pernisine was purified using affinity chromatography and gel-exclusion chromatography, as presented schematically in Fig 2A. The chromatogram for the purified pernisine (Fig 2B) and the SDS-PAGE of selected pernisine fractions showed the purified pernisine at around 55 kDa (Fig 2C, red line).

The constructs containing the *pernisine* ^*wt*^ gene were not successfully overexpressed in any of the tested expression cells (i.e., *E*. *coli* BL21(DE3), BL21(DE3)pLysE and BL21(DE3)pMagic, BL21-CodonPlus(DE3)RIL), as none of the expressed protein was seen at significantly higher levels compared to the cell lysates before and after induction, using SDS-PAGE (S2 Fig). The BL21(DE3) cell growth curve obtained by measuring OD_600_ did not show any significant deviation compared to the control BL21(DE3) cells (transformed with empty pMCSG7; data not shown), which indicated that the recombinant pernisine is non-toxic for *E*. *coli*.

While *E*. *coli* is the most used host for heterologous gene expression, sometimes codon use indicates that it is not an optimal host for expression of the recombinant proteins because of the significantly divergent codon bias between the two organisms, especially for the first 10 codons at the beginning of the translation [23]. As a consequence, for heterologous gene expression, the presence of non-optimal codons in the DNA sequence expressed can result in inefficient translation, and sometimes in aborted translation. Using the BL21(DE3)pMagic and BL21-CodonPlus(DE3)RIL *E*. *coli* strain carrying a plasmid for rare tRNA aminoacyls (e.g., Arg [AGG], Arg [AGA], Ile [AUA], Leu [CUA]), we replaced the rarest codons for pernisine, as given in S1 Table. However, supplying these extra tRNAs did not resolve the problem of producing pernisine at detectable levels using SDS-PAGE.

Catara and co-workers overexpressed recombinant *pernisine* ^*wt*^ that lacked the signal sequence in *E*. *coli*, but could not detect it distinctively in crude extracts using SDS-PAGE [1]. They observed the recombinant pernisine indirectly, through its degradation products.

We compared the expression of *pernisine* ^*wt*^ and *pernisine* ^*co*^ and *pernisine* ^*S355Aco*^ in the pMCSGx constructs using the BL21(DE3) *E*. *coli* strain. Initially, small-scale expression was carried out to evaluate the time of expression for pernisine production. First, dot blots of the total cells lysates at prolonged growth times after induction (1, 2, 3, 4 h) showed that 3 h after induction was optimal for *pernisine* ^*co*^ overexpression (Fig 2D) and again no His_6_-tagged protein using the pernisine^wt^ sequence was detected. Later, large-scale expression was performed as described in the Materials and methods. Analysis of the cell lysates by Western blotting revealed overexpressed pernisine^co^ at around 55 kDa, 100 kDa and 80 kDa (Fig 2F, lanes 2, 4, 6), which represented pernisine with the His_6_ tag, fusion with MBP, and fusion with the GST tag, respectively. Also here pernisine^wt^ could not be detected (Fig 2F, lanes 1, 3, 5). The protein lysates of pernisine^co^ and the mutant pernisine^S355Aco^ showed these overexpressed protein bands, as marked with black arrows in Fig 2E (lanes 1–6), at the molecular weights corresponding with the Western blotting. From the SDS-PAGE analysis of the pellet, we estimated that around 20% of the recombinant pernisine was insoluble (data not shown). Addition of the MBP or GST tags did not significantly improve the pernisine^co^ solubility or overexpression in *E*. *coli*. TEV cleavage efficiency was 88% in the case without fusion partners. Whereas fusion partners (GST and MBP tag) resulted in about two times lower efficiency (data not shown). The apparent molecular weights of the pernisine^co^ fused with the tags were higher than the theoretical molecular weights (S2 Table). The reason for this is most likely the physical nature of the recombinant pernisine itself. The shape and charge of proteins have effects on their mobility under SDS-PAGE. Altered SDS stoichiometry can result in electrophoretic anomalies, as shown for highly hydrophobic proteins or their parts [24], as can incomplete denaturation of the pernisine before electrophoresis. The final yields of the lyophilised pernisine^co^ and pernisine^S355Aco^ were around 10 mg per litre of culture. Indeed, the use of such codon-optimised genes is becoming more attractive, and recently more examples of improved overexpression of such proteins have been reported [25,26,27]. Although there have been further studies carried out, to date, improvements to heterologous protein expression using codon-optimisation or by supplying extra tRNAs remain more or less empirical [28].

### Identification and maturation of recombinant pernisine

Recombinant pernisine was detected by immunodetection (Fig 2D and 2E) and by N-terminal sequencing and MS/MS analysis. Immunodetection showed distinctively the codon-optimised His_6_-tagged pernisine (Fig 2D and 2E). N-terminal sequencing showed that the purified recombinant pernisine cleaved with TEV protease starts with S-N-A-A-A. Those five aa represent the remaining residues from the TEV cleavage site of the tagged pernisine. The recombinant pernisine at an apparent molecular weight of 55 kDa represents its preform, and includes the His_6_ tag (Fig 1A, lanes 1, 2, mark I). During temperature maturation in the presence of CaCl_2_, the pernisine underwent autoproteolytic cleavage of its N-terminal proregion, and the resulting mature pernisine was seen at around 36 kDa (Fig 1A, lanes 1, 2, mark II). The theoretical mass of the putative signal sequence plus the proregion (1–92 aa) of the recombinant pernisine was around 9.3 kDa. The addition of a fusion tag modifies this by *ca*. 2 kDa. The apparent molecular mass of the recombinant pernisine was around 8 kDa above the theoretical molecular mass. MS/MS investigation of the N- and C-terminals of the selected matured and non-matured pernisine indicated that the cleavage site of the proregion appears to be between Gln92 and Ala93 (S3 and S4 Figs). Indeed, a comparison of S3 and S4 Figs shows higher abundance of the peptides identified from the pernisine N-terminal to aa 92. The abundance of the peptides from aa 93 to the pernisine C-terminal is more or less the same across S3 and S4 Figs These data are in agreement with the SDS-PAGE analysis and the prediction of the native pernisine proregion defined from its alignment with Tk-subtilisin [2]. The purified pernisine was dissolved in 10 mM HEPES, pH 8.0, with 1 mM CaCl_2_, and it was activated for 1 h at 90°C, as determined as the optimised conditions in the azocasein assay. The mutation of Ser355 into Ala (S355A) did not affect the process of pernisine maturation, as seen in Fig 1A, comparing lanes 1 and 2, but have resulted in a complete activity inhibition of pernisine, comparing lanes 1 and 2 or after heat activation lanes 3 and 4 (Fig 1B). That supports the thesis that Ser355 is a nucleophile involved in a catalytic triade. Thermally induced maturation is already known for other proteases [29], and Ca^2+^ is important for enzyme stability at higher temperatures [1,2,30]. Binding sites for Ca^2+^ are one of the general adaptations of thermostable enzymes. Such bound Ca^2+^ increases the thermostability of the subtilases or protects them from autolysis [30].

### Biochemical characterisation of recombinant pernisine

The enzymatic activity of the activated pernisine was determined qualitatively using zymography and quantitatively using the azocasein assay, unless otherwise indicated. Before the enzymatic assays were carried out, the pernisine was heat activated in 10 mM HEPES, pH 8.0, with 1 mM CaCl_2_ at 90°C for 1 h, as seen for Fig 1B, comparing lanes 1 and 3. Only wild-type of recombinant pernisine was used in further studies.

#### The effects of CaCl_2_ and/or NaCl on proteolytic activity

Fig 2G illustrates the relative activities of recombinant pernisine evaluated using the azocasein assay (92°C, 20 min, pH 8.0), according to increasing CaCl_2_ concentrations from 0 mM to 32 mM. The maximum enhanced relative activity of the recombinant pernisine was observed at 1 mM CaCl_2_. Further increases in the CaCl_2_ concentration led to a gradual decrease in the pernisine relative activity. The effects of increasing NaCl concentrations from 0 to 500 mM were also investigated for the relative activity of the heat-activated recombinant pernisine (90°C, 1 h, pH 8.0) both in the absence and presence of 1 mM CaCl_2_. Relative activity of recombinant pernisine is enhanced in the range from 100 to 300 mM NaCl. It is likely that higher ionic strength induces favourable surface-surface electrostatic interactions that are especially important for thermostability of proteins [31]. Pernisine without heat activation was also tested as a negative control (Fig 2H). These data indicate that this pernisine heat activation is Ca^2+^ dependent, but is not affected by increased ionic strength with NaCl.

#### Effects of pH and temperature on enzymatic activity

**Fig 3:**
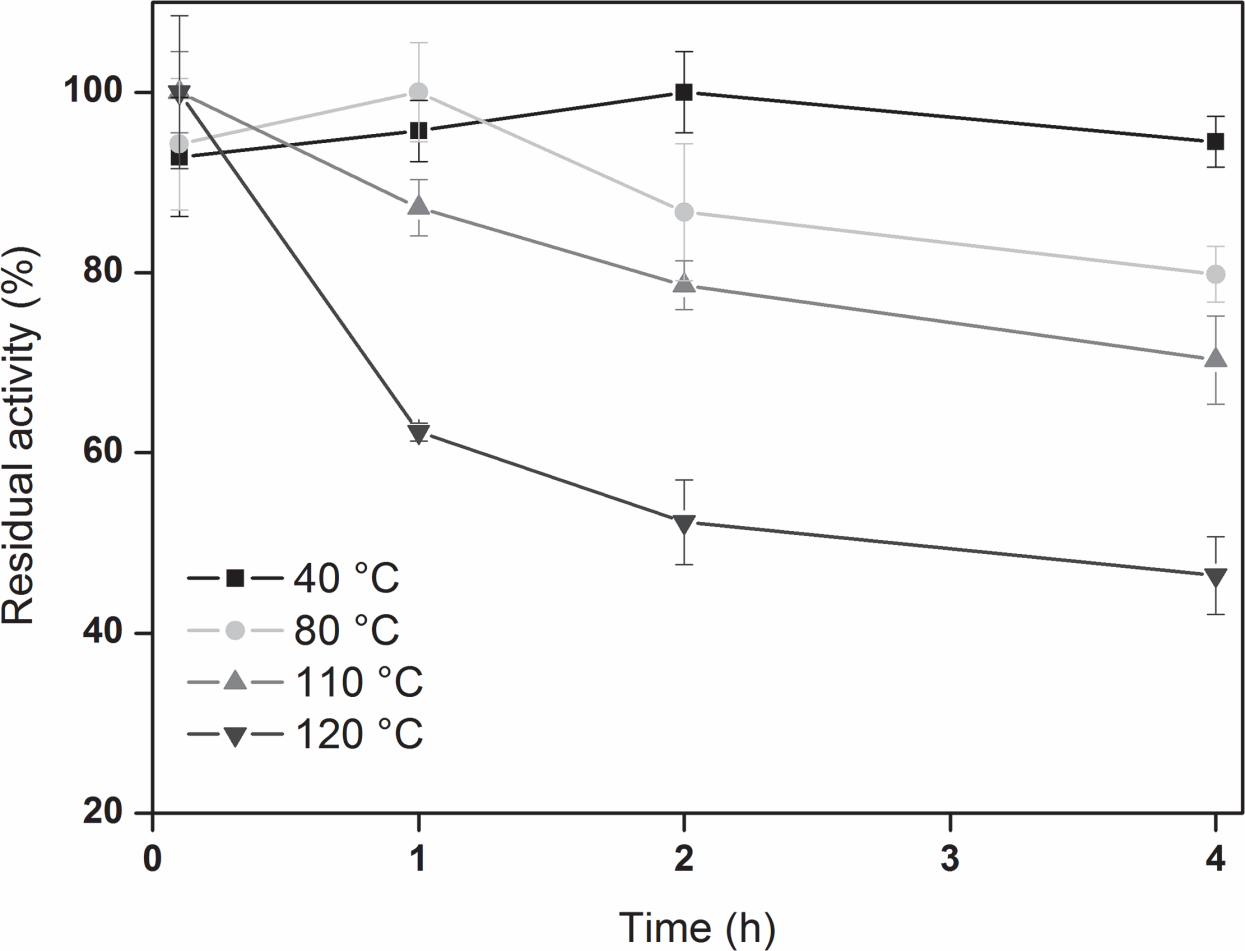
Thermostability of recombinant pernisine^co^. Time courses of the residual activity of recombinant pernisine at the indicated temperatures, at pH 8.0.

The enzyme activities of recombinant pernisine at different temperatures (40, 80, 110, 120°C) at pH 7.6 (±0.6) for prolonged incubation times (12, 60, 120, 240 min) were also investigated. This revealed that the pernisine enzymatic activity was retained to at least 80°C (Fig 3). Also, after a 4-h incubation at 120°C, pernisine still retained 40% of its activity.

**Fig 4:**
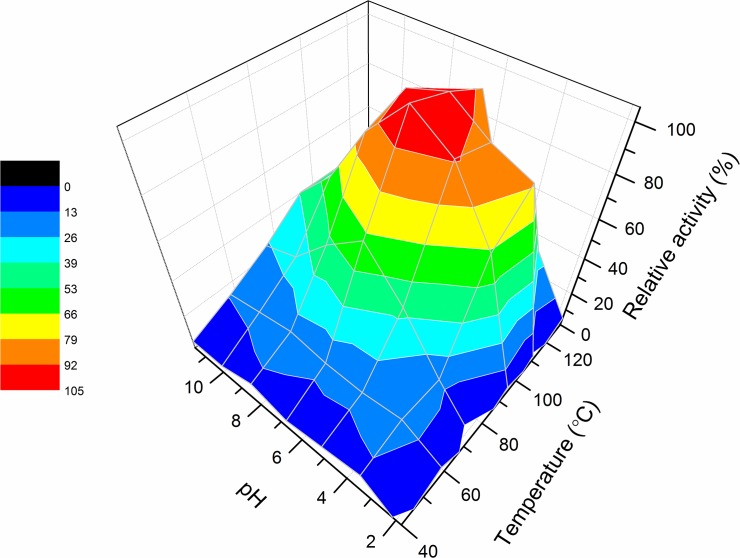
Dependence of the activity of recombinant pernisine on temperature and pH. Relative activity of recombinant pernisine according to temperature and pH (as corrected based on temperature change). Assays were carried out in triplicates, and means of the relative activity dependence are presented as a function of temperature and corrected pH. Colour legend on the left indicates the relative activities.

To investigate the full variability of this pernisine activity, the pH values were corrected according to the temperature change factors (dpH dT^-1^). As illustrated in Fig 4, the three-dimensional representation of the dependence of the pernisine relative activity on temperature and pH shows that recombinant pernisine shows more than 90% relative activity in the pH range from 4.5 to 9.1 and in the temperature range from 90°C to 110°C, with its maximum activity at pH 7.0 and 100°C.

Thus, this recombinant pernisine retains equivalent enzymatic performance under these temperature and pH conditions as the native pernisine, which has shown optimum activity at pH 6.8 and 105°C [2].

#### Effect of inhibitors and denaturing agents on the enzymatic activity

Different protease inhibitors and denaturing agents were also tested for their effects on the enzymatic activity of recombinant pernisine, as evaluated using azocasein assays. Inhibitors of metallo, serine and cysteine proteases were included in the repertoire, as specified in Table 1. As expected, the greatest inhibitory effects on this pernisine activity were seen for PMSF, EDTA and EGTA, which confirms that this recombinant pernisine is properly folded serine protease that is modulated by Ca^2+^. EDTA and EGTA are chelators of Ca^2+^, which, as expected, resulted in inhibition of the recombinant pernisine activity. Iodoacetamide did not have any relevant effect on this enzymatic activity. Table 2 gives the effects of the various reductants, denaturants and detergent on the enzymatic activity of recombinant pernisine. The effects of the two reductants (Table 2, DTT, 2-mercaptoethanol) on the recombinant pernisine activity at 1 mM and 5 mM each were similar, with residual pernisine activities of 58% and 48%, and 51% and 38%, respectively. In contrast, the presence of the denaturants (Table 2, guanidine-HCl, urea) resulted in increased pernisine enzymatic activity above the control. With the addition of 0.1% and 3% SDS, the recombinant pernisine activity showed 9% and 90% inhibition, respectively. The enzymatic activities of recombinant pernisine in the presence of these reductants, denaturants and detergent showed less inhibition compared to those of the native pernisine [2]. The reason for this might arise from the different pathway in the pernisine maturation process. The native pernisine was matured *in vivo* and was seen as a 36-kDa band as well as a *ca*. 23-kDa band, which might have resulted from its further processing [2]. In contrast, the recombinant pernisine was matured *in vitro* and was just seen as a 36-kDa band. Indeed, there remains the possibility that the maturation process of the recombinant pernisine is not fully complete or optimise.

**Table 1:** Residual protease activities of recombinant pernisine in the presence of the protease inhibitors.

**Addition**	**Concentration**	**Residual proteinase activity at 1 mM CaCl**_2_ **(%)**	
	**(mM)**	**The present study**	**Šnajder et al (2012) [2]**
None	/	100.0 ±1.7	100.0
EDTA	1	92.8 ±0.6	93.5
	5	2.1 ±0.1	0.5
EGTA	1	90.5 ±5.5	91.2
	5	8.2 ±1.0	1.4
PMSF	1	6.1 ±0.8	6.9
	10	0.9 ±0.5	2.8
IAA	1	69.1 ±6.5	ND
	10	89.6 ±2.0	91.7

ND, no data.

**Table 2:** Residual protease activities of recombinant pernisine in the presence of the reductants, denaturants and detergent.

**Reagent**	**Concentration**	**Residual proteinase activity at 1 mM CaCl**_2_ **(%)**	
		**The present study**	**Šnajder et al (2012) [2]**
None	/	100 ±4.7	100.0
DTT	1 mM	58.0 ±2.3	27.9
	5 mM	47.9 ±4.5	25.9
2-MeEtOH	1%	50.6 ±11.6	34.8
	5%	38.4 ±2.5	30.8
Guanidine-HCl	1 M	124.9 ±4.0	66.2
	4 M	189.6 ±10.1	152.2
Urea	1 M	119.7 ±9.7	51.7
	4 M	106.0 ±3.1	46.8
SDS	0.10%	90.8 ±0.8	65.7
	3%	10.4 ±2.8	33.8

Thus, this recombinant pernisine retains an equivalent enzymatic performances against specified inhibitors and better enzymatic performances against denaturing agents as the native pernisine.

## Conclusions

In the present study, we have shown that codon optimisation is a key step for the successful expression of pernisine in *E*. *coli* from a distant host like Archaea. With codon optimisation using the Genscript algorithm we replaced the codons that are rare for the host with more frequent ones, and we minimised any unfavourable mRNA structures during the translation. This resulted in increased expression levels (up to 10 mg L^-1^), making this recombinant pernisine a potential product for industry. Furthermore, we have shown that mutation of the pernisine aa sequence at the catalytic site (S355A) leads to a complete loss of pernisine activity, as expected. This recombinant pernisine has an N-terminal proregion that is autocleaved during maturation in the presence of CaCl_2_.

## Supporting Information

S1 Fig Variations in the DNA sequences of wild-type and codon-optimised pernisine. The predicted aa sequence of the 43 kDa pernisine is indicated as capital letters. Variations in the DNA sequences are shown in bold letters. The functional domains represent the signal sequence (underlined) and the proregion (double underlined), with the mature pernisine representing the rest of the sequence. The aa involved in the predicted catalytic triad are coloured in yellow (Asp149 [D149], His184 [H184], Ser355 [S355]). The start codon is marked with “*”, the stop codon with “–“. Note that only ATG was used as a start codon for the heterologous expression.
(TIF)

S2 Fig SDS-PAGE analysis and zymography of the overexpression of the pernisine^wt^. Representative gels of the cell lysates from the overexpression of pernisine^**wt**^, following electrophoresis on standard 12% SDS-PAGE (A) and on 12% SDS-PAGE with casein as substrate (B) for the zymography activity (4 h at 80°C). Staining was with Coomassie blue dye. Lanes 0, protein MW markers (indicated left); lanes 1, pMCSG7-pernisine^wt^ before induction; lanes 2, pMCSG7-pernisine^wt^; lanes 3, pMCSG9-pernisine^wt^ and 4, pMCSG10-pernisine^wt^. BL21-CodonPlus(DE3)RIL cells were grown at 37°C until OD_600_ of 0.6.The induction condition was 1 mM IPTG at 37°C for 3 h.
(TIF)

S3 Fig MS/MS analysis of the N-terminal and C-terminal of pernisine^co^ with the proregion (band I). Individual identified peptides are aligned with the pernisine sequence. Top: Schematic representation of the structure of the pernisine signal sequence (1–24 aa) and proregion (25–92 aa), and of the mature pernisine (93–430 aa).
(DOCX)

S4 Fig MS/MS analysis of N-terminal and C-terminal of mature pernisine^co^ (band II). Individual identified peptides are aligned with the pernisine sequence. Top: schematic representation of mature pernisine.
(DOCX)

S1 Table Codon distribution of the wild-type and codon-optimised pernisine sequences. (DOCX)

S2 Table Expression vectors used in this study. (DOCX)

## References

[1] Catara G, Ruggiero G, La Cara F, Digilio FA, Capasso A, Rossi M (2003) A novel extracellular subtilisin-like protease from the hyperthermophile *Aeropyrum pernix* K1: biochemical properties, cloning, and expression. Extremophiles 7: 391–399. 12908102 10.1007/s00792-003-0337-4

[2] Šnajder M, Vilfan T, Černilec M, Rupreht R, Popović M, Juntes P, et al (2012) Enzymatic degradation of PrP^Sc^ by a protease secreted from *Aeropyrum pernix* K1. PLoS ONE 7: e39548 10.1371/journal.pone.0039548 22761822 PMC3386259

[3] Koga Y, Tanaka S-i, Sakudo A, Tobiume M, Aranishi M, Hirata A, et al (2014) Proteolysis of abnormal prion protein with a thermostable protease from Thermococcus kodakarensis KOD1. Appl Microbiol Biotechnol 98: 2113–2120. 10.1007/s00253-013-5091-7 23880875

[4] Hirata A, Hori Y, Koga Y, Okada J, Sakudo A, Ikuta K, et al (2013) Enzymatic activity of a subtilisin homolog, Tk-SP, from *Thermococcus kodakarensis* in detergents and its ability to degrade the abnormal prion protein. BMC Biotechnol 13: 1–7. 10.1186/1472-6750-13-1 23448268 PMC3599501

[5] Sako Y, Nomura N, Uchida A, Ishida Y, Morii H, Koga Y, et al (1996) *Aeropyrum pernix* gen. nov., sp. nov., a novel aerobic hyperthermophilic Archaeon growing at temperatures up to 100°C. Int J Syst Bacteriol 46: 1070–1077. 8863437 10.1099/00207713-46-4-1070

[6] Siezen RJ, Leunissen JAM (1997) Subtilases: the superfamily of subtilisin-like serine proteases. Protein Sci 6: 501–523. 9070434 10.1002/pro.5560060301 PMC2143677

[7] Palmieri G, Cannio R, Fiume I, Rossi M, Pocsfalvi G (2009) Outside the unusual cell wall of the hyperthermophilic archaeon *Aeropyrum pernix* K1. Mol Cell Proteomics 8: 2570–2581. 10.1074/mcp.M900012-MCP200 19640852 PMC2773722

[8] Wang Y, Zhang Y-HP (2009) Overexpression and simple purification of the *Thermotoga maritima* 6-phosphogluconate dehydrogenase in *Escherichia coli* and its application for NADPH regeneration. Microb Cell Fact 8: 30 10.1186/1475-2859-8-30 19497097 PMC2701922

[9] Terpe K (2006) Overview of bacterial expression systems for heterologous protein production: from molecular and biochemical fundamentals to commercial systems. Appl Microbiol Biotechnol 72: 211–222. 16791589 10.1007/s00253-006-0465-8

[10] Welch M, Govindarajan S, Ness JE, Villalobos A, Gurney A, Minshull J, et al (2009) Design parameters to control synthetic gene expression in *Escherichia coli* . PLoS ONE 4: e7002 10.1371/journal.pone.0007002 19759823 PMC2736378

[11] Elena C, Ravasi P, Castelli ME, Peirú S, Menzella HG (2014) Expression of codon optimized genes in microbial systems: current industrial applications and perspectives. Front Microbiol 5. 10.3389/fmicb.2014.00021 PMC3912506 24550894

[12] Wacker M, Linton D, Hitchen PG, Nita-Lazar M, Haslam SM, North SJ, et al (2002) N-Linked glycosylation in *Campylobacter jejuni* and its functional transfer into *E*. *coli* . Science 298: 1790–1793. 12459590 10.1126/science.298.5599.1790

[13] Burgess-Brown NA, Sharma S, Sobott F, Loenarz C, Oppermann U, Gileadi O (2008) Codon optimization can improve expression of human genes in *Escherichia coli*: a multi-gene study. Protein Expr Purif 59: 94–102. 10.1016/j.pep.2008.01.008 18289875

[14] Menzella H (2011) Comparison of two codon optimization strategies to enhance recombinant protein production in *Escherichia coli* . Microb Cell Fact 10: 15 10.1186/1475-2859-10-15 21371320 PMC3056764

[15] Song H, Li G, Mai W, Huang G, Chen K, Zhou J, et al (2014) Codon optimization enhances protein expression of *Bombyx mori* nucleopolyhedrovirus DNA polymerase in *E*. *coli* . Curr Microbiol 68: 293–300. 10.1007/s00284-013-0476-5 24129839

[16] Milek I, Cigić B, Skrt M, Kaletunç G, Ulrih NP (2005) Optimization of growth for the hyperthermophilic archaeon *Aeropyrum pernix* on a small-batch scale. Can J Microbiol 51: 805–809. 16391661 10.1139/w05-060

[17] Eschenfeldt WH, Lucy S, Millard CS, Joachimiak A, Mark ID (2009) A Family of LIC Vectors for High-Throughput Cloning and Purification of Proteins In: Doyle S, editor. High Throughput Protein Expression and Purification: Humana Press pp. 105–115. 10.1007/978-1-59745-196-3_7 PMC2771622 18988021

[18] Bradford MM (1976) A rapid and sensitive method for the quantitation of microgram quantities of protein utilizing the principle of protein-dye binding. Anal Biochem 72: 248–254. 942051 10.1016/0003-2697(76)90527-3

[19] Beynon RJ, Easterby JS (1996) Buffer solutions Oxford: IRL Press at Oxford University Press viii, 84 p. p.

[20] Gvritishvili AG, Leung KW, Tombran-Tink J (2010) Codon preference optimization increases heterologous PEDF expression. PLoS ONE 5: e15056 10.1371/journal.pone.0015056 21152082 PMC2994832

[21] Wang Q, Mei C, Zhen H, Jess Z (2012) Codon preference optimization increases prokaryotic cystatin C expression. J Biomed Biotechnol vol. 2012: 7. 10.1155/2012/732017 PMC3471025 23093857

[22] Terpe K (2003) Overview of tag protein fusions: from molecular and biochemical fundamentals to commercial systems. Appl Microbiol Biotechnol 60: 523–533. 12536251 10.1007/s00253-002-1158-6

[23] Goodman DB, Church GM, Kosuri S (2013) Causes and effects of N-terminal codon bias in bacterial genes. Science 342: 475–479. 10.1126/science.1241934 24072823

[24] Rath A, Glibowicka M, Nadeau VG, Chen G, Deber CM (2009) Detergent binding explains anomalous SDS-PAGE migration of membrane proteins. Proc Natl Acad Sci U S A 106: 1760–1765. 10.1073/pnas.0813167106 19181854 PMC2644111

[25] Hu H, Gao J, He J, Yu B, Zheng P, Huang Z, et al (2013) Codon optimization significantly improves the expression level of a keratinase gene in *Pichia pastoris* . PLoS ONE 8: e58393 10.1371/journal.pone.0058393 23472192 PMC3589435

[26] Volontè F, Piubelli L, Pollegioni L (2011) Optimizing HIV-1 protease production in *Escherichia coli* as fusion protein. Microb Cell Fact 10: 53 10.1186/1475-2859-10-53 21718537 PMC3141379

[27] Zylicz-Stachula A, Zolnierkiewicz O, Sliwinska K, Jezewska-Frackowiak J, Skowron PM (2014) Modified 'one amino acid-one codon' engineering of high GC content TaqII-coding gene from thermophilic *Thermus aquaticus* results in radical expression increase. Microb Cell Fact 13: 7 10.1186/1475-2859-13-7 24410856 PMC3893498

[28] Angov E, Legler PM, Mease RM (2011) Adjustment of Codon Usage Frequencies by Codon Harmonization Improves Protein Expression and Folding In: Evans JTC, Xu M-Q, editors. Heterologous Gene Expression in *Ecoli*: Humana Press pp. 1–13. 10.1007/978-1-61737-967-3_1 21125377

[29] Pulido M, Saito K, Tanaka S, Koga Y, Morikawa M, Takano K, et al (2006) Ca^2+^-dependent maturation of subtilisin from a hyperthermophilic archaeon, *Thermococcus kodakaraensis*: the propeptide is a potent inhibitor of the mature domain but is not required for its folding. Appl Environ Microbiol 72: 4154–4162. 16751527 10.1128/AEM.02696-05 PMC1489632

[30] Smith CA, Toogood HS, Baker HM, Daniel RM, Baker EN (1999) Calcium-mediated thermostability in the subtilisin superfamily: the crystal structure of *Bacillus Ak*.1 protease at 1.8 A resolution. J Mol Biol 294: 1027–1040. 10588904 10.1006/jmbi.1999.3291

[31] Dominy BN, Minoux H, Brooks CL (2004) An electrostatic basis for the stability of thermophilic proteins. Proteins 57: 128–141. 15326599 10.1002/prot.20190

