# Supplementary figures and images for: Codon Optimisation Is Key for Pernisine Expression in Escherichia coli
Source: PLoS One. 2015 Apr 9;10(4):e0123288. doi: 10.1371/journal.pone.0123288 (PMC4391949; doi:10.1371/journal.pone.0123288)

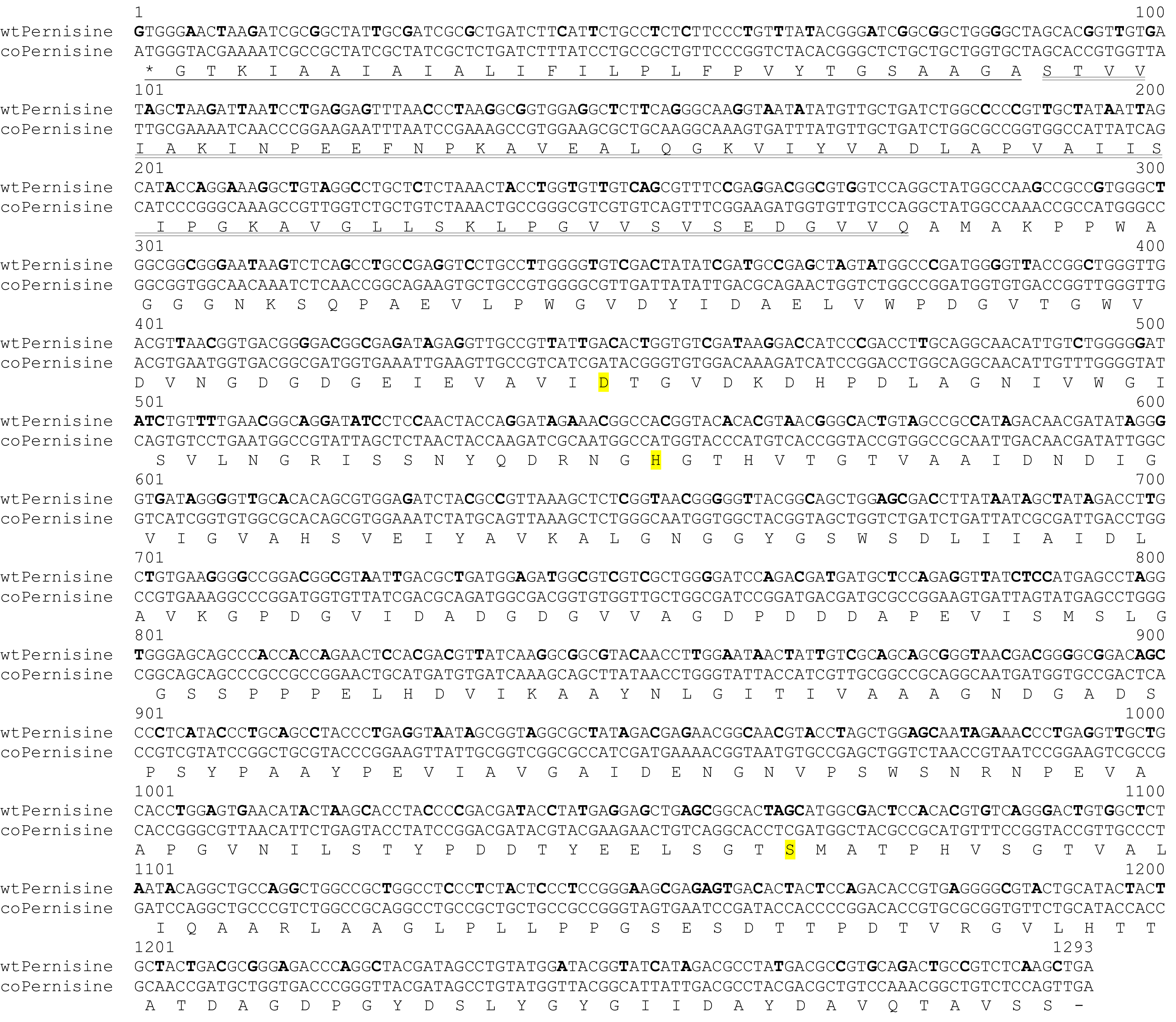

Supplement: S1 Fig — The predicted aa sequence of the 43 kDa pernisine is indicated as capital letters. Variations in the DNA sequences are shown in bold letters. The functional domains represent the signal sequence (underlined) and the proregion (double underlined), with the mature pernisine representing the rest of the sequence. The aa involved in the predicted catalytic triad are coloured in yellow (Asp149 [D149], His184 [H184], Ser355 [S355]). The start codon is marked with “*”, the stop codon with “–“. Note that only ATG was used as a start codon for the heterologous expression. (TIF) [file pone.0123288.s001.tif]

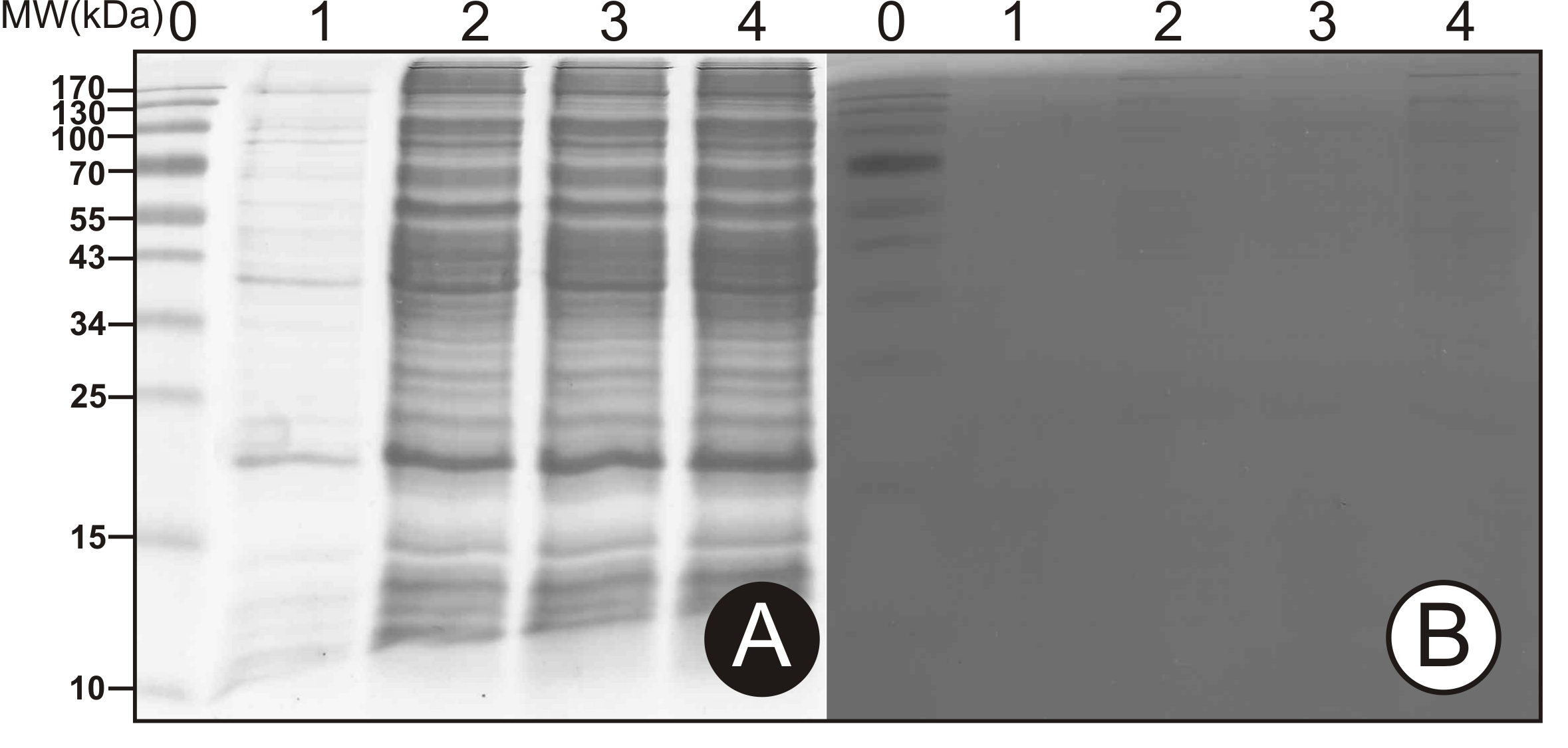

Supplement: S2 Fig — Representative gels of the cell lysates from the overexpression of pernisinewt, following electrophoresis on standard 12% SDS-PAGE (A) and on 12% SDS-PAGE with casein as substrate (B) for the zymography activity (4 h at 80°C). Staining was with Coomassie blue dye. Lanes 0, protein MW markers (indicated left); lanes 1, pMCSG7-pernisinewt before induction; lanes 2, pMCSG7-pernisinewt; lanes 3, pMCSG9-pernisinewt and 4, pMCSG10-pernisinewt. BL21-CodonPlus(DE3)RIL cells were grown at 37°C until OD600 of 0.6.The induction condition was 1 mM IPTG at 37°C for 3 h. (TIF) [file pone.0123288.s002.tif]

**Supporting Information Figure S3**


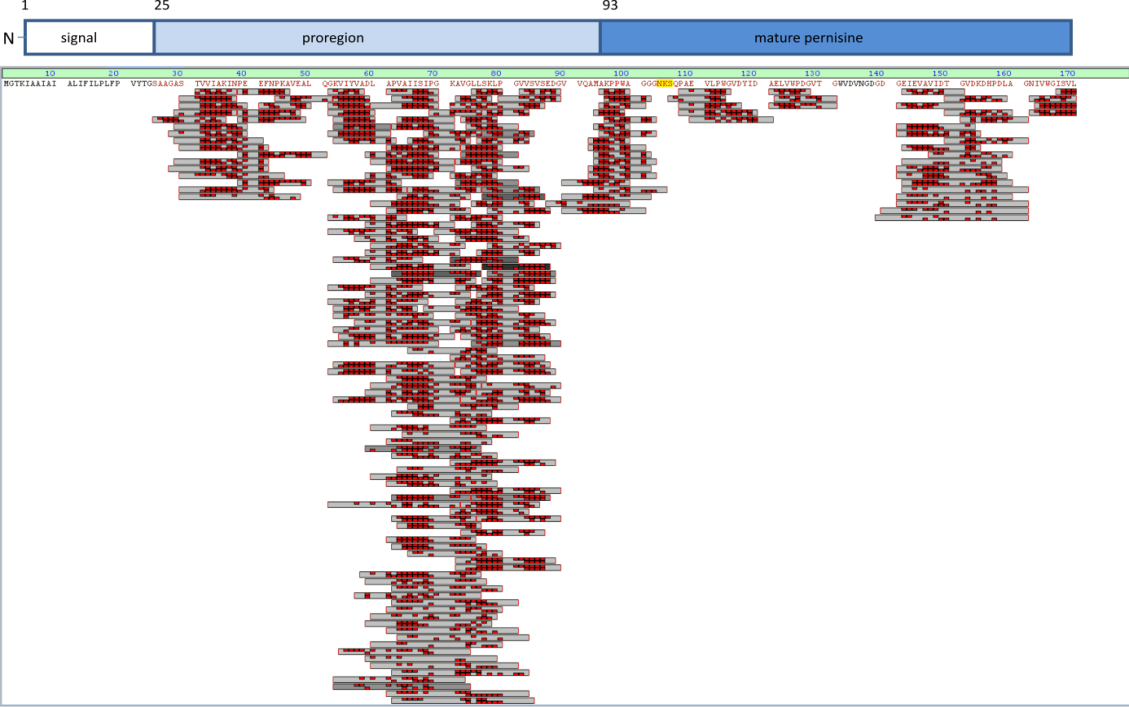

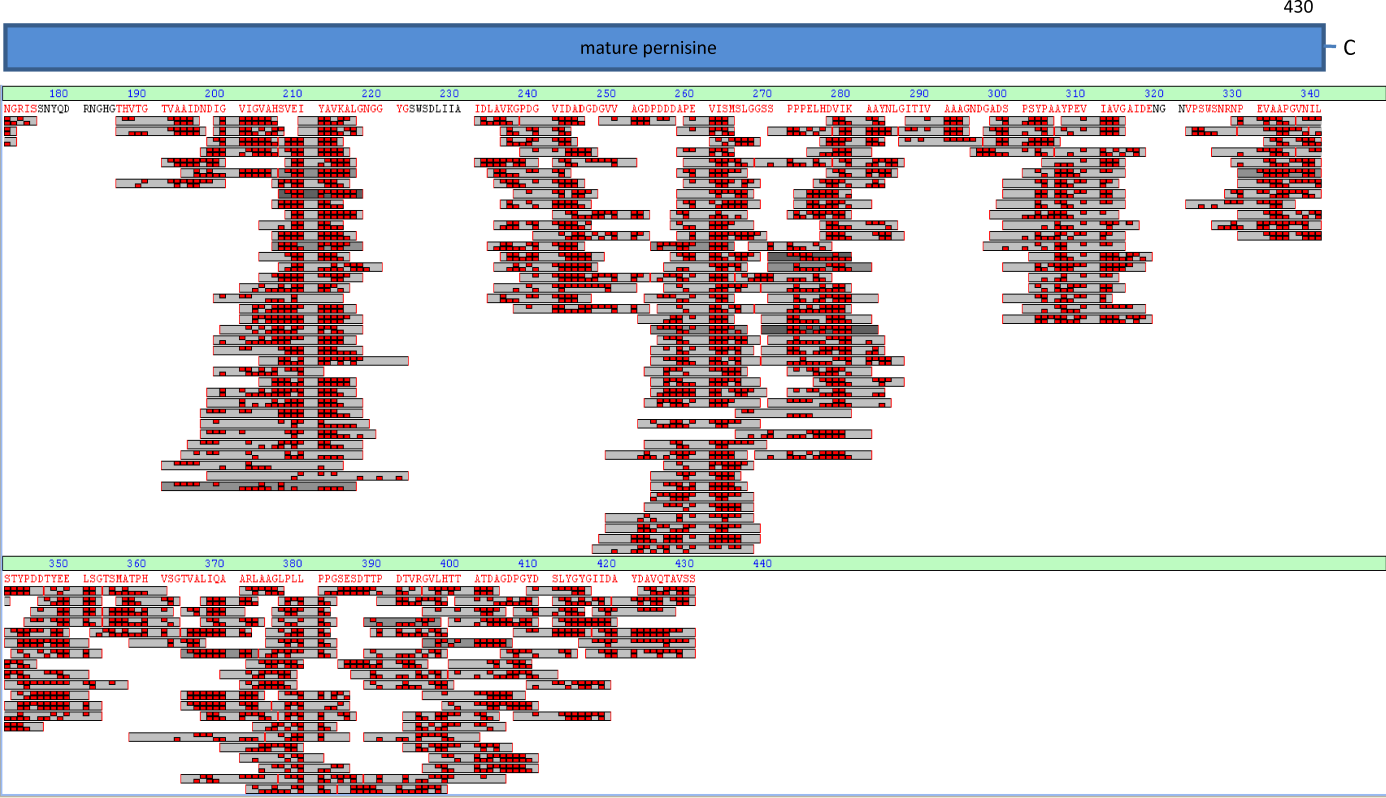

Supplement: S3 Fig — Individual identified peptides are aligned with the pernisine sequence. Top: Schematic representation of the structure of the pernisine signal sequence (1–24 aa) and proregion (25–92 aa), and of the mature pernisine (93–430 aa). (DOCX) [file pone.0123288.s003.docx]

**Supporting Information Figure S4**


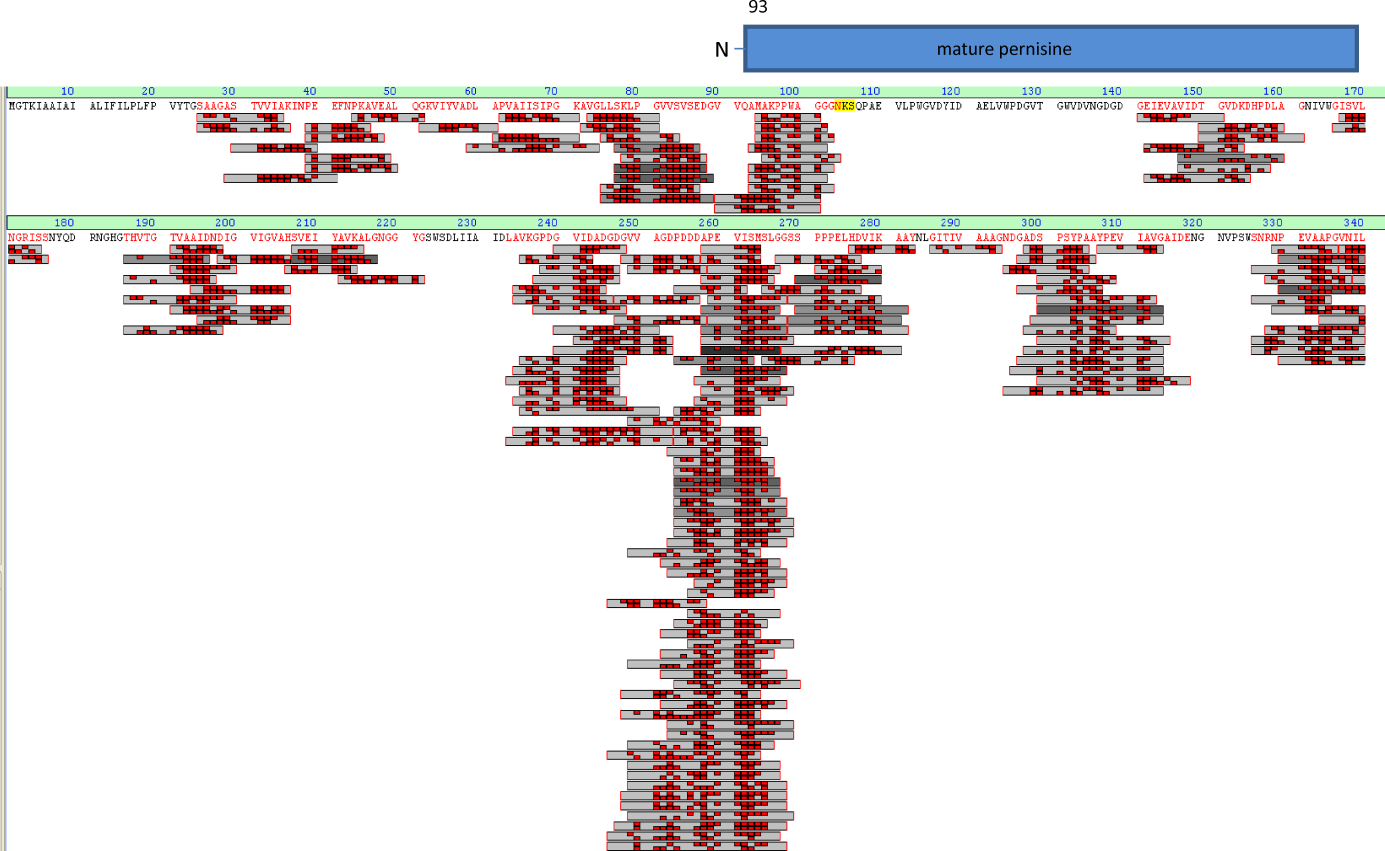

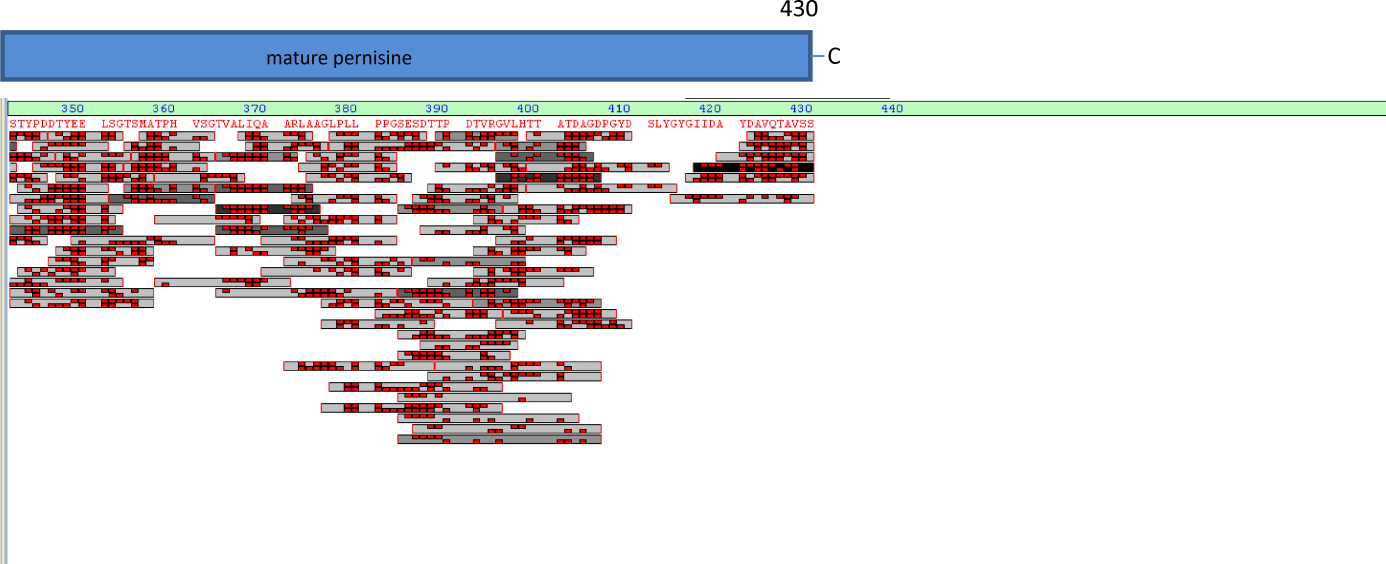

Supplement: S4 Fig — Individual identified peptides are aligned with the pernisine sequence. Top: schematic representation of mature pernisine. (DOCX) [file pone.0123288.s004.docx]
