# Supplementary material for: Codon Optimisation Is Key for Pernisine Expression in Escherichia coli
Source: PLoS One. 2015 Apr 9;10(4):e0123288. doi: 10.1371/journal.pone.0123288 (PMC4391949; doi:10.1371/journal.pone.0123288)
Supplement: S1 Table — (DOCX) [file pone.0123288.s005.docx]

**S1 Table:** Codon distribution of the wild-type and codon-optimised pernisine sequences.

| **Codon** | **Amino acid** | **Fraction^1^** | **Pernisine** | |  | **Codon** | **Amino acid** | **Fraction^1^** | **Pernisine** | |
| --- | --- | --- | --- | --- | --- | --- | --- | --- | --- | --- |
|  |  |  | **wt** | **co** |  |  |  |  | **wt** | **co** |
| TAA | * | 0.61 | 0 | 0 |  | TTA | L | 0.14 | 0 | 0 |
| TAG | * | 0.09 | 0 | 0 |  | TTG | L | 0.13 | 1 | 0 |
| TGA | * | 0.30 | 1 | 1 |  | CTT | L | 0.12 | 5 | 0 |
| GCT | A | 0.18 | 22 | 17 |  | CTC | L | 0.10 | 6 | 0 |
| GCC | A | 0.26 | 14 | 17 |  | CTA | L | 0.04 | 6 | 0 |
| GCA | A | 0.23 | 6 | 11 |  | CTG | L | 0.47 | 9 | 27 |
| GCG | A | 0.33 | 12 | 9 |  | AAA | K | 0.74 | 2 | 12 |
| CGT | R | 0.36 | 0 | 3 |  | AAG | K | 0.26 | 10 | 0 |
| CGC | R | 0.36 | 0 | 2 |  | ATG | M | 1 | 3 | 4 |
| CGA | R | 0.07 | 0 | 0 |  | TTT | F | 0.58 | 1 | 2 |
| CGG | R | 0.11 | 0 | 0 |  | TTC | F | 0.42 | 2 | 1 |
| AGA | R | 0.07 | 2 | 0 |  | CCT | P | 0.18 | 14 | 0 |
| AGG | R | 0.04 | 3 | 0 |  | CCC | P | 0.13 | 5 | 0 |
| AAT | N | 0.49 | 3 | 8 |  | CCA | P | 0.20 | 9 | 1 |
| AAC | N | 0.51 | 14 | 9 |  | CCG | P | 0.49 | 4 | 31 |
| GAT | D | 0.63 | 13 | 22 |  | TCT | S | 0.17 | 3 | 6 |
| GAC | D | 0.37 | 25 | 16 |  | TCC | S | 0.15 | 4 | 3 |
| TGT | C | 0.46 | 0 | 0 |  | TCA | S | 0.14 | 3 | 3 |
| TGC | C | 0.54 | 0 | 0 |  | TCG | S | 0.14 | 1 | 3 |
| CAA | Q | 0.34 | 0 | 4 |  | AGT | S | 0.16 | 1 | 5 |
| CAG | Q | 0.66 | 6 | 2 |  | AGC | S | 0.25 | 18 | 10 |
| GAA | E | 0.68 | 1 | 17 |  | ACT | T | 0.19 | 13 | 0 |
| GAG | E | 0.32 | 16 | 0 |  | ACC | T | 0.40 | 4 | 15 |
| GGT | G | 0.35 | 10 | 27 |  | ACA | T | 0.17 | 1 | 0 |
| GGC | G | 0.37 | 20 | 25 |  | ACG | T | 0.25 | 3 | 6 |
| GGA | G | 0.13 | 9 | 0 |  | TGG | W | 1 | 7 | 7 |
| GGG | G | 0.15 | 13 | 0 |  | TAT | Y | 0.59 | 6 | 7 |
| CAT | H | 0.57 | 2 | 6 |  | TAC | Y | 0.41 | 9 | 8 |
| CAC | H | 0.43 | 5 | 1 |  | GTT | V | 0.28 | 17 | 18 |
| ATT | I | 0.49 | 8 | 17 |  | GTC | V | 0.20 | 10 | 12 |
| ATC | I | 0.39 | 8 | 18 |  | GTA | V | 0.17 | 10 | 0 |
| ATA | I | 0.11 | 19 | 0 |  | GTG | V | 0.35 | 12 | 18 |

^1^Fraction of relative use of each codon in its synonymous codon family

* stop codon
