# Supplementary material for: Codon Optimisation Is Key for Pernisine Expression in Escherichia coli
Source: PLoS One. 2015 Apr 9;10(4):e0123288. doi: 10.1371/journal.pone.0123288 (PMC4391949; doi:10.1371/journal.pone.0123288)
Supplement: S2 Table — (DOCX) [file pone.0123288.s006.docx]

**S2 Table:** Expression vectors used in this study.

| **Vector** | **Leader sequence** | **MW (kDa)** |
| --- | --- | --- |
| pMCSG7 | N-His_6_-TEV-Pernisine^wt/co^ | 46.5 |
| pMCSG9 | N-His_6_-MBP-TEV-Pernisine^wt/co^ | 87.4 |
| pMCSG10 | N-His_6_-GST-TEV-Pernisine^wt/co^ | 72.8 |
